# Cellular and Animal Models of Striated Muscle Laminopathies

**DOI:** 10.3390/cells8040291

**Published:** 2019-03-29

**Authors:** Hannah A. Nicolas, Marie-Andrée Akimenko, Frédérique Tesson

**Affiliations:** 1 Department of Biology, Faculty of Science, University of Ottawa, Ottawa, ON K1N 6N5, Canada; hnico019@uottawa.ca (H.A.N.); makimen@uottawa.ca (M.-A.A.); 2 Interdisciplinary School of Health Sciences, University of Ottawa, Ottawa, ON K1N 6N5, Canada

**Keywords:** lamin A/C, striated muscle laminopathies, DCM, EDMD, L-CMD, LGMD, cellular models, animal models

## Abstract

The lamin A/C (*LMNA*) gene codes for nuclear intermediate filaments constitutive of the nuclear lamina. *LMNA* has 12 exons and alternative splicing of exon 10 results in two major isoforms—lamins A and C. Mutations found throughout the *LMNA* gene cause a group of diseases collectively known as laminopathies, of which the type, diversity, penetrance and severity of phenotypes can vary from one individual to the other, even between individuals carrying the same mutation. The majority of the laminopathies affect cardiac and/or skeletal muscles. The underlying molecular mechanisms contributing to such tissue-specific phenotypes caused by mutations in a ubiquitously expressed gene are not yet well elucidated. This review will explore the different phenotypes observed in established models of striated muscle laminopathies and their respective contributions to advancing our understanding of cardiac and skeletal muscle-related laminopathies. Potential future directions for developing effective treatments for patients with lamin A/C mutation-associated cardiac and/or skeletal muscle conditions will be discussed.

## 1. Introduction

Laminopathies consist of a variety of diseases that affect different tissue types ranging from cardiac muscle (e.g., dilated cardiomyopathy (DCM)), skeletal muscles (e.g., Emery-Dreifuss Muscular Dystrophy (EDMD)), adipose tissues (e.g., familial partial lipodystrophy Dunnigan-type 2(FPLD2)) to nervous system (e.g., Charcot-Marie-Tooth disease type 2B1 (CMT2B1)) or affect several systems (e.g., Hutchison Gilford Progeria Syndrome (HGPS)). Mutations in the nuclear A-type lamins coding gene *LMNA* cause primary (classical) laminopathies, while secondary laminopathies are caused by mutations in B-type lamins, proteins involved in prelamin A maturation, or lamin binding partners. In addition, Lamin A/C mutations are also associated with arrhythmogenic right ventricular cardiomyopathy and hypertrophic cardiomyopathy [1,2,3,4,5]. The *LMNA* gene (approximately 24 kb) is located on chromosome 1q22 and has 12 exons. It is expressed in most differentiated cells, with the expression in mice starting during early embryogenesis (8th day post-implantation) [6,7]. Alternative splicing of exon 10 results in the generation of the two major lamin splice variants, A and C [6]. Lamins A and C are identical up until amino acid (a.a) 566, after which six lamin C specific a.a are added [8]. Prelamin A is further processed to produce mature lamin A, firstly by the farnesylation of the cysteine residue in the C-terminal *CAAX* motif [9]. This is followed by the removal of the *AAX* a.a in the motif, then the methylation of the farnesylated cysteine, and lastly by the cleavage of the last 15 a.a (including the cysteine that was both methylated and farnesylated), resulting in a 646 a.a-long mature lamin A [9]. The other isoforms of lamin A/C are lamin C2 (utilizing an alternative exon 1; found in mammalian sperm cells) and lamin AΔ10 (which lacks exon 10; a less abundant form) [10,11,12]. The A-type lamins possess three main parts: the carboxy (C) head and amino (N) tail involved in the assembly of lamin A/C homodimers into anti-parallel protofilaments, and the central rod domain involved in the formation of the lamin A/C homodimers [13]. The A-type lamins, which are nuclear intermediate filaments, have a range of functions including nuclear structural support, anchoring of nuclear pores, chromatin and protein binding [14]. Examples of A-type lamins’ binding partners are the sterol regulatory element binding transcription factor 1 (SREBF-1), the LEM-domain integral membrane protein emerin, and the spectrin repeat-containing nuclear envelope protein 1 (syne1), in which both emerin and syne1 are involved in mediating anchorage to the cytoskeleton [15,16,17,18].

The mechanism(s) by which the same *LMNA* mutation can sometimes lead to different phenotypes and mutations found throughout the ubiquitously expressed gene can lead to tissue specific phenotypes is not yet well elucidated. However, based on the current data, three hypotheses seek to explain this diversity in phenotypes. The first suggests that the accumulation of misprocessed/unprocessed lamin A is toxic to cells, as in HGPS [19,20]. The second relates to the role of lamin A/C in interacting with chromatin and proteins. This is evidenced, for instance, by the increased expression and activation of MAPK pathway members in EDMD and DCM [21]. Similarly, the most frequent mutation causing FPLD2 affects a lamin A/C domain critical for binding of SREBF-1, a transcription factor (TF) involved in modulating transcription of sterol-regulated genes and adipocyte differentiation [15,22,23,24,25,26,27,28]. Lastly, it was postulated that the disruption of the protein network that connects the nucleus to the rest of the cell results in exacerbated effects of external stimuli such as mechanical forces on myocytes, leading to aberrant cellular structure and response (e.g. disruption of the cytoskeleton and cellular mechanical and adhesion properties in cells expressing the DCM lamin A/C p.D192G variant) [29,30,31,32,33,34,35,36].

The majority of reported cases of laminopathies (79%) affect the cardiac and/or skeletal muscles and are predominantly autosomal dominant [37]. DCM associated with *LMNA* mutations accounts for up to at least one-third of reported genotyped families with DCM, and presents with dilation of the left ventricle, thinning of the ventricular wall and conduction defects [38]. Patients with *LMNA*-related DCM also tend to have more severe phenotype and poorer prognosis with a recent study reporting 19% required heart transplants [39,40]. *LMNA*-related congenital muscular dystrophy (L-CMD) and EDMD are skeletal muscle conditions caused by *LMNA* mutations with overlapping clinical phenotypes, which include wasting of the upper arm and lower front leg muscles [41]. However, L-CMD is more severe and has earlier onset but less definitive cardiac presentation compared to EDMD [42]. 1B Limb-girdle muscular dystrophy (LGMD1B), associated with *LMNA* mutations, also affects the skeletal and cardiac muscles [43]. It is characterized by progressive muscle weakness, age-related cardiac conduction defects and no or minimal late-onset joint contractures [43]. With the first paper identifying *LMNA* as a causal gene in EDMD in 1999, massive efforts involving both cellular and animal models have since been undertaken to understand the underlying mechanisms in cardiac and skeletal muscle conditions caused by mutations in *LMNA* [6,44]. Thus, this review will examine the different phenotypes observed in these established models and their respective contributions to unravelling the mechanisms underlying the development of striated muscle laminopathies. Potential future directions for developing more effective treatments for patients with *LMNA* mutation-associated conditions will also be discussed. 

## 2. Cellular Models

Cellular models facilitate the characterization of the striated muscle laminopathy phenotype at both the cellular and nuclear level, and the identification of potential molecular markers for the conditions. This section gives an overview of the results obtained using patients’ and mice models’ cells and various cell lines.

### 2.1. Evidence of Abnormal Nuclear Morphology Coupled with Aberrant Lamin A/C Phenotype and Mislocalization of Several Lamin A/C Binding Proteins in LMNA-Related Cardiac and Skeletal Muscle Disease

A rare lamin A/C null case in humans was discovered from a deceased newborn who was homozygous for the nonsense p.Y259X mutation [45]. This variant causes complete absence of the A-type lamins in the homozygous state and half the amount of wild-type (WT) lamin A/C in the heterozygous state, resulting in LGMD1B [45,46]. The homozygous child presented with severe under development and abnormalities including dystrophic muscles [45]. Skin fibroblasts from the heterozygous grandmother were comparable to WT controls, while the *LMNA* null cells presented with misshapen nuclei and abnormal localization of nuclear proteins such as lamin B1, B2, emerin, and syne1 [46]. B-type lamins are intimately connected to A-type lamins, and appear to be involved in several cellular functions ranging from regulation of gene expression and mitotic spindle assembly to cellular senescence [47]. Moreover, the skin fibroblasts from the child had impaired cell division capacity [46]. RNAi knockdown of *LMNA* in HeLa cells, however, did not affect cell growth but indeed resulted in emerin mislocalization in the endoplasmic reticulum, similar to what was seen in the *LMNA* null fibroblasts [46,48]. To determine whether emerin was also mislocalized in the presence of other *LMNA* mutations, mutant lamins A and C were transfected in various cell lines including the P19 embryonic carcinoma stem cells, which do not have endogenous lamin A/C [30,49]. The results were contradictory and, regardless of the position of the mutation along *LMNA*, emerin was either clearly mislocalized in some studied DCM or EDMD mutant lamin A/C or normally expressed and distributed [30,49,50]. Nonetheless, many *LMNA* mutations affecting the rod domain of the protein and causing muscular laminopathies also presented with aggregates of lamin A/C proteins, particularly lamin C, in the nucleoplasm of transfected cells [49,50]. Emerin was found in these nuclear foci [50]. On the other hand, a.a substitutions affecting the globular end of the lamin A/C protein did not result in punctate lamin A structures and emerin remained mislocalized [50]. Analyses of fibroblasts from muscular laminopathy patients with mutations affecting the head or tail domains of the protein had overt nuclear structure aberrations [51]. Specifically, immunostaining for lamin A/C revealed defects including honeycomb staining, punctate structures or blebbing [51]. Moreover, emerin and lamin B were also noted to be mislocalized [51]. All of these results therefore demonstrate that lamin A/C mutations cause profound nuclear abnormalities and the mislocalization of a variety of proteins. 

Transient transfection in HeLa cells of the p.E203G lamin A variant, which is associated with DCM with conduction defects (DCM-CD), resulted in lamin aggregates and the absence of the lamin A variant in the nuclear periphery [15]. However, Ostlund and colleagues did not show this effect when they transiently transfected C2C12 cells with the p.E203G prelamin A variant [52,53]. The difference between the observed lamin phenotypes might be attributed to the cell line used. Nevertheless, the transient expression (lamin A only, lamin C only or both lamins A and C (to maintain the 1:1 stoichiometry of lamin A/C)) of other variants for DCM (p.D192G, p.N195K) or EDMD (p.R386K) in Cos7 and H9C2 (rat cardiomyoblast cell line) cells resulted in large and multiple lamin aggregates, which also fail to connect to the nuclear envelope unlike cells expressing WT or an FPLD (p. R482W) mutant lamin A/C [54]. An interesting exceptionwas the DCM-causing lamin A/C variant p.L85R, which produced a WT lamin phenotype if mutant lamin A only was expressed but an aberrant phenotype (multiple speckles) when mutant lamin C only was expressed; nevertheless, the abnormal mutant lamin C phenotype was rescued if co-expressed with the mutant lamin A [54]. This therefore suggests that mutations can differently affect the properties of lamins A and C. Indeed, interactions between emerin and lamin A, and emerin and lamin C, are differently affected by *LMNA* mutations [55]. Furthermore, fibroblasts from mice expressing only lamin C (*Lmna*^LCO^) (discussed in the later part of the review) displayed normal emerin localization [56]. These results illustrate that the structural abnormalities due to *LMNA* mutations can result in the inability of the A-type lamins to properly connect to the nuclear envelope and can negatively impact their interaction with their binding partners. To further explore the effects of *LMNA* mutations on the interaction between A-type lamins and their binding partners, studies involving mutations found in regions known to interact with lamin A/C partners were performed.

### 2.2. Evidence of Disrupted Lamin A/C Interaction with Binding Partners, which Can Result in Multiple Tissue Phenotypes

Mutations found throughout the *LMNA* gene affect sites that are known to interact with various proteins. An example of one of these mutations—the substitution of C with T at nucleotide position 1444, producing the p.R482W lamin A/C variant and causing both FPLD and LGMD in some patients—is located in a region known to bind actin and/or influence SREBF1 interaction [15,57,58,59,60]. Similar to p.L530P, an EDMD variant, the p.R482W FPLD variant showed a notable decrease in binding capacity to SREBF1, thus suggesting that in some mutations that cause striated muscle laminopathies, binding capacity to TF involved in adipocyte metabolism can be affected, which can then lead to lipodystrophic features in some patients with primary presentation of muscular laminopathy [15]. This could also explain why the *Lmna*^Δ8–11^ mice, which primarily exhibited striated muscle laminopathy, also notably lacked subcutaneous fat and presented with some metabolic parameter abnormalities [61,62]. To further investigate the consequences of changes in lamin A/C structure and the resulting abnormal lamin A/C phenotypes conferred by the mutations, mobility experiments were performed.

### 2.3. Evidence of Impaired Lamin A/C Structural Stability and Dynamics in Striated Muscle Laminopathies

As a consequence of the change in lamin A/C structure caused by *LMNA* mutations, the structural stability and dynamics of various lamin A/C variants were studied. Gilchrist et al. transfected epitope-tagged WT lamin A or lamin A variants in HT1080 cells, a human fibrosarcoma cell line [63]. The lamin A variants studied were p.L85R, p.N195K (both associated with DCM-CD), p.L530P (associated with EDMD) and p.R482W (associated with FPLD in some individuals co-presenting LGMD) [63]. The transfected cells showed no significant or overt nuclear shape/size abnormalities [63]. Fluorescence recovery after photobleaching (FRAP) analysis was performed on these transiently transfected cells to determine the dynamics of WT lamin A and variant lamin A. The DCM-CD and EDMD variants were more mobile and showed faster dynamics (especially the p.N195K variant) compared to WT [63]. However, the predominant lipodystrophy causing variant p.R482W had comparable mobility to WT lamin A [63]. FRAP experiments on Cos7 cells also demonstrated increased mobility for the p.D192G (DCM-CD) and p.R386K (EDMD) lamin C only expressing variants compared to either WT lamin C or the p.L85R (DCM-CD) lamin C variant [54]. Fluorescence loss in photobleaching (FLIP) experiments further showed faster diffusion of lamin A p.N195K compared to WT [63]. Such increased mobility suggests less stability in the lamin A/C structures of the said variants [54,63]. To determine if these unstable A-type lamins affect the mechanical properties of the nucleoskeleton and/or the cytoskeleton, studies were performed on WT and mutant *LMNA*-expressing cells. 

### 2.4. Evidence of Compromised Nuclear and Cytoskeletal Mechanics in the Presence of LMNA Mutations Causing Muscular Laminopathies

**Figure 1:**
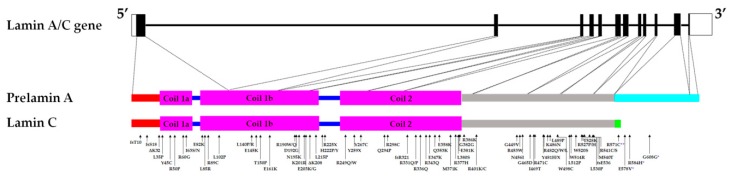
Schematic of prelamin A and lamin C proteins with the corresponding coding exons of the *LMNA* gene and the positions of the affected amino acids cited in this review. In red is the N terminus head, in bright pink are coils 1a, b and 2, in blue are linkers 1 and 2, in grey is the part of the C terminal tail common to both lamins A and C, in cyan is the part of the C terminal tail specific to lamin A and in bright green is the part of the C terminal tail specific to lamin C. fs means frameshift starting at the indicated amino acid. * and ** denote lamin-A- or lamin-C-specific amino acid substitution respectively.

Putative alterations of the nucleoskeleton and/or the cytoskeleton were examined by assessing their mechanical properties in the presence of *LMNA* mutations. A cardiac sample from the p.D192G DCM patient presented with immense nuclear aberrations including rupture of nuclear envelope, chromatin disorganization and mislocalization of the mitochondria in the nucleoplasm in about 30% of the cardiomyocytes [38,64]. Similarly, nuclear envelope breaks were observed in the *Lmna*^ΔK32/+^ DCM mice model (discussed later part in the review) [65]. Furthermore, skin fibroblasts from DCM, EDMD and LGMD patients carrying *LMNA* mutations displayed a loss of nuclear stiffness [30]. Mouse embryo fibroblasts (MEFs) and skeletal myotubes from the *Lmna*^Δ8–11^ mice (discussed later in the review) also presented with reduced nuclear stiffness and a notable increase in nuclear deformity when strain was applied to the cells compared to WT [31,32,33]. Both low- and medium-pressure micro-injection of dextran into the nucleus resulted in dextran leaking into the cytoplasm, suggesting compromised nuclear integrity in fibroblasts of *Lmna*^Δ8–11^ mice [31]. Viral transfection of different mutant *LMNA* cDNA (p.E161K, p.D192G, p.N195K variants) causing DCM with or without conduction defects in neonatal rat ventricular myocytes, resulted in nuclear morphology defects including blebbing [34]. Analyses of nuclear stiffness showed that the mutant *LMNA*-expressing cells are stiffer, particularly the p.D192G variant, compared to the controls [34]. On the other hand, *LMNA* mutations that were not associated with striated muscle laminopathies did not result in nuclear deformability [30]. Nucleoplasmic localization and large aggregates of lamin A/C were described in transfected cells, suggesting impaired assembly of lamin A/C mutants [30,34,38,54]. These mutations are found in the rod domain of the A-type lamins, which is involved in dimerization and the formation of a higher-order lamin structure (Figure 1).

Moreover, although no overt differences were detected between the cytoskeletal architecture of WT and *Lmna*^Δ8–11^ MEFs, a significant decrease in cytoskeletal stiffness, cytoplasmic elasticity and viscosity was observed in the *Lmna*^Δ8–11^ fibroblasts compared to WT cells, indicating that lamin A/C regulates the cytoskeleton plasticity [31,32]. Even skin fibroblasts with EDMD mutations that did not result in nuclear deformability displayed defects in force transmission between the nucleus and the cytoskeleton [30]. Neonatal rat ventricular myocytes expressing mutant *LMNA* showed disorganized actin and lower levels of actin staining compared to WT and uninfected controls [34]. They also have impaired adhesion and increased plasticity, suggesting a poorer capacity to handle and transmit mechanical stress due to actin defects [34]. Accordingly, left ventricular *Lmna*^Δ8–11^ myocytes displayed contractile dysfunction [66]. Actin and the p38 MAPK, which was shown to be associated with *LMNA*-related DCM, are thought to interact, and the inhibition of p38 restored the mutant actin and mechanical phenotypes back to WT [34,67,68]. Defects in mechano-transduction in *LMNA* mutant cells have been further explored using L-CMD patients’ myoblasts cultured on matrix stiffness close to that of muscle (12 kPa) [35,36]. Mutant cells displayed accumulation and disorganization of contractile actin stress fibres and increased traction forces, supporting the hypothesis that mutant cells are unable to sustain high external mechanical stretching because of impaired functional integrity of nuclear–cytoskeletal linkages [35,36]. Furthermore, *LMNA*-mutated myoblasts showed aberrant activation of regulators of the mechano-response (including yes-associated protein involved in cell adaptation to its microenvironment and formins known to affect actin polymerisation and depolymerisation in a force-sensitive manner) [35,36]. Interestingly, nuclear shape and chromatin organization can be improved in lamin A/C-depleted U2OS cells (derived from human osteosarcoma) in HGPS-derived patients cells, or in aged vascular smooth muscle cell by treatment with remodelin [69,70]. Remodelin is a small molecule that inhibits N-Acetyltransferase 10 (NAT10) activity to induce a microtubule reorganization [70]. These results emphasize the significance of nuclear structural deformity in laminopathies, demonstrating that mutations in *LMNA* impair the mechanical capacity and integrity of both the nucleus and the whole cell by the disruption of the cytoskeleton, thus resulting in defects in mechano-transduction signalling. To determine if these structural changes also affect the post-translational modification status of lamin A/C, experiments looking at such were undertaken.

### 2.5. Evidence of Altered Lamin A/C Post-Translational Modification Status in Striated Muscle Laminopathy

As discussed in the previous paragraphs, many of the disease-causing mutations result in alteration of the lamin A/C protein structure as well as the nuclear envelope properties. Therefore, would such changes in conformation also result in aberrant post-translational protein modifications? A-type lamins are known to be phosphorylated and the phosphorylation status may influence nuclear deformability [71]. Lamin A/C phosphorylation determines the nuclear localization, assembly, and plays a role in mobility and cytoplasmic transport of nuclear lamins during interphase [72]. There are three main regions of lamin A that are phosphorylated during interphase: the N-terminal head, the proximal C-terminal region and the far C-terminal region found in lamin A only [72]. Mitsuhashi et al. demonstrated abnormal Akt phosphorylation of the serine residue at position 458 (located in the proximal C-terminal region) in muscle tissues and fibroblasts of patients with myopathies caused by *LMNA* mutations located in the Ig fold in the protein C-terminus [73]. Resolved structure of the lamin A/C C-terminus showed that in WT, S458 was partially buried; therefore, it was plausible that certain mutations in the Ig fold altered the protein structure and caused the exposure of S458, rendering it accessible for abnormal phosphorylation by Akt [73,74]. Moreover, the EDMD variant p.R453W not only increased S458 phosphorylation, but also decreased S390 phosphorylation as compared to WT lamin A [75]. In addition, N-terminus lamin A phosphorylation was found to be lower in myoblasts from EDMD and LGMD patients compared to the control [76]. Furthermore, immunostaining for phosphorylated lamin A/C (phos-lamin A/C) in muscle fibres from these patients was markedly reduced [76]. Lower phos-lamin A/C was also observed in the regenerating muscle of a patient with EDMD compared to a sample from a patient with another type of muscular dystrophy, Duchenne Muscular Dystrophy (DMD) [76]. Such reduction in phos-lamin A/C was only observed in myocytes but not in fibroblasts and inflammatory cells, suggesting a tissue-specific process involving kinases and lamin A/C in muscles [76]. Overall, the data showed how a mutation that affects a particular region of the protein may confer structural and chemical changes that result in a molecular hallmark common to the different types of *LMNA*-related myopathies.

Lamin A has also been shown to be sumoylated by SUMO2 [77]. Fibroblasts from a p.E203K DCM patient or HeLa-transfected cells showed decreased lamin A sumoylation and increased cell death [77]. Moreover, transient transfection of p.D192G lamin C only or both p.D192G lamins A and C in fibroblast-like Cos7 cells derived from monkey kidney tissue or in C2C12 cells, a mouse skeletal myoblast cell line, resulted in large lamin foci, which co-localized with SUMO1, a protein involved in post-translational modification [38,78]. Mislocalization of SUMO1 was also observed in *Lmna*^H222P/H222P^ mice’s (discussed later in the review) primary myoblasts and in *Lmna*^H222P/H222P^ mice’s skeletal muscle tissue [78]. These results therefore suggest that lamin A sumoylation impairment is involved in the disease mechanism underlying striated muscle laminopathies. 

Overall, the data from patient cells and immortalized cell lines demonstrate the wide breadth of the effects caused by mutations in the lamin A/C gene. Nonetheless, such cellular phenotypic analyses can be further characterized using derived cells from the patients’ induced pluripotent stem cells (iPSCs). These iPSCs can be of immense benefit, particularly in cases when multiple tissue phenotypes are co-present, as there will be no need to collect multiple cell types from the patients. Some studies using laminopathy patient iPSCs are discussed in the next section.

### 2.6. Derived Myogenic or Cardiac Cells from Patients’ Induced iPSCs Replicated Previously Demonstrated Muscular Laminopathy Cellular Phenotypes with 3D Culturing: A Promising Method of Identifying and Examining More Subtle Morphological Defects in Mutant Cells

In primary culture, dermal fibroblasts from the p.R225X lamin A/C DCM-CD patient presented with decreased lamin A/C and profound nuclear defects including condensed heterochromatin clumps, nuclear blebs, nuclear pore complexes (NPCs) clustering, and mitochondria around the nuclear envelope [79]. Electrical induction of these fibroblasts resulted in a significant increase in the senescence and apoptosis in the mutant cells; inhibition of the ERK 1/2 branch of the MAPK pathway ameliorated this phenotype [79]. Cardiomyocytes were then derived from the iPSCs obtained from the fibroblasts of the p.R225X patient and another DCM-CD patient with a different lamin A/C truncation [79,80]. Surprisingly, both the mutant cardiomyocytes were morphologically comparable to WT [79]. However, upon electrical stimulation, these mutant cardiomyocytes presented with nuclear senescence and a marked increase in apoptosis [79]. The electrical susceptibility of these cardiomyocytes seems to be caused by lamin A/C haploinsufficiency as electrical stimulation of *LMNA* knocked-down control cells resulted in the same phenotype as the mutant cells [79]. Another study using derived myogenic cells from the iPSCs of patients with various striated muscle laminopathies (p.K32del, p.L35P and p.R249W) presented with deformed nuclei and aberrant localization of emerin (localized in one pole of the cells) and lamin B1 [81]. Lamins A and C also formed aggregates or displayed a honeycomb pattern [81]. Although an abnormal nuclear shape (observed in p.L35P and p.R249W cells) and mislocalized lamin A/C (observed in p.R249W cells) were noted in the myotubes (differentiated myoblasts), the phenotype tended to be less pronounced in the myotubes than in the myoblasts [81]. Interestingly, 3D culturing of these mutant myotubes, which better simulate the cellular environment in patients, enhanced the phenotypes observed compared to what was seen in the monolayer-cultured myotubes [81]. These data therefore highlight the immense potential of iPSCs to model striated laminopathies and the promising use of 3D culturing as a means to detect, identify and more accurately characterize cellular changes that might not be readily observable in 2D culturing.

## 3. Animal Models

In order to further confirm the pathogenicity of certain *LMNA* mutations and to study the phenotypes and tease out disease-associated pathways while minimizing confounding variables such as comorbidity, defined and controlled strains of different animal models have been used to study striated laminopathies. 

### 3.1. Caenorhabditis elegans (Worm) Models

#### Worm Models Mimicked Patient Cellular Phenotypes, Highlighted the Role of Lamin A/C in Germ Cells and Reproduction, and Further Confirmed Results from Cell Lines and Mice Models

The worm species *Caenorhabditis elegans* (*C. elegans*) possesses one lamin gene (*lmn-1*) that codes for lamin-1, the equivalent of the B-type lamins but functionally similar to both A and B type lamins [82]. Gonadal knockdown of *lmn-1* via RNAi resulted in abnormal embryos that are developmentally arrested and have abnormal nuclear morphology and disrupted chromatin [83]. Animals that survived lmn-1 knockdown and grew to adulthood were either sterile or semi-sterile [83]. The sterile animals presented with a significant reduction of germ cells, some of which showed abnormalities [83]. Similarly, meiotic arrest and apoptosis were observed in *Lmna*^Δ8–11^ male mice germ cells (however, oogenesis in mice was unaffected) [84]. Moreover, siRNA knockdown of *Lmna* in the seminiferous tubules of mice resulted in sperm with abnormal heads [85]. Nevertheless, the progenies of the semi-sterile adult *C. elegans* were fertile and had an increased male proportion compared to WT [83]. NPCs were also misarranged in the nuclei of embryonic cells with decreased lamin-1, and such defects have been observed in cell lines, other animal models and patients [45,61,83,86]. Expression of GFP-tagged p.K46del lamin-1 variant, an equivalent of the p.K32del EDMD/L-CMD human lamin A/C variant, also resulted in lamin misassembly, nuclear aggregates, emerin mislocalization (due to decreased binding to lamin) and muscle/movement abnormalities [87,88,89]. Further evaluation of 14 laminopathic variants (including EDMD and DCM variants) located throughout the protein showed abnormal homodimer assembly, as well as mislocalization and abnormal dynamics of lamin-1 and/or aberrant mislocalization/dynamics and even lethality in vivo [90]. Moreover, as in cells transfected with certain lamin A/C variants, emerin was found in some of the lamin-1 variant aggregates [50,90]. The expression of the p.Y59C EDMD lamin-1 variant led to tissue-specific muscle promoter inactivation, coupled with aberrant muscle gene expression changes, resulting in muscle disruption and decreased muscle function [91].

### 3.2. Drosophila melanogaster (Fruit Fly) Models

#### Fruit Fly Models Phenocopied Early Lethality in LMNA Null Patients, Demonstrated Cytosketal Disturbance, Presented with Muscle and Mobility Defects, and Associated the Oxidative/Reductive Stress Signalling Pathway (via Nrf2) to Striated Muscle Laminopathies

Similar to the *lmn-1* gene of *C. elegans*, the *Drosophila melanogaster LamC* gene encoding lamin-C is also a paralogue of the lamin A/C gene in vertebrates [92]. *LamC* null fruit fly and truncated Lamin-C variants fruit flies (deletion of the first 42 or 48 N-terminal residues) were generated and resulted in developmental delay and prepupal death [93,94]. Larval muscle-specific expression of the 42 a.a. N-terminal truncated lamin C variant, however resulted in some viability but the survivors presented with deformed legs in adulthood, caused by the absence of muscular function and the disturbance of ecdysone (moulting) hormone signalling [94]. Immunostaining for the fruit fly B-type lamin (lamin Dm0) in both the null and the N-terminal deleted mutants showed lamin B nuclear aggregation [93]. On the other hand, ectopic overexpression of a 9 a.a truncated lamin C variant resulted in formation of melanotic tumours, lag in growth, inhibition of pupation and morbidity at the larval stage [95]. In the *LamC* null fruit fly, localization of B-type lamins and heterochromatin was normal, but NPC clustering was observed [96]. Furthermore, nuclear aberrations including chromatin leakage were detected in the cells of imaginal discs of *LamC* null larvae compared to WT [96]. Abnormal nuclear shape and actin localization in the nucleus were also seen in muscle samples from *LamC* null flies [96].

Expression of a variety of EDMD and DCM *LMNA* variants in fruit fly resulted in a host of nuclear aberrations such as lamin-C aggregates and/or increased nuclear blebbing, which are similar to what were previously observed in transfected cells expressing striated muscle laminopathy mutations [53,93,96]. C-terminal and N-terminal truncated lamin-C expression also both resulted in nuclear abnormalities [96]. In addition, when the N-terminal truncated lamin-C or the p.W557S (human equivalent: p.W520S) variant was expressed either ubiquitously or in a muscle-specific manner, lethality was observed [96]. Evaluation of nuclear stiffness in WT and mutant lamin-C demonstrated increased nuclear fragility in the 48 a.a. N-terminal truncated lamin C variant, further illustrating the importance of the lamin N-terminus in muscles [97]. Muscle-specific expression of another set of muscular laminopathy mutations resulted in larval muscular and mobility defects, actin disruption, nuclear protein mislocalization in the cytoplasm and varying degrees of mortality in mutant pupae [97,98]. Transcriptome analyses from mutant and WT body muscle wall samples showed elevated expression levels of detoxification genes in mutant samples compared to WT [97]. An increase in the TF Nrf2 involved in activating detoxification genes was observed, along with an increase in p62/SQSTM1, an autophagosome cargo protein involved in capturing Keap1, a protein that sequesters Nrf2 [97]. The release of Nrf2 from Keap1 allows Nrf2 to translocate to the nucleus, where it upregulates genes coding for antioxidant proteins [97]. Muscle biopsies from striated muscle laminopathy patients presented with increased staining for p62/SQSTM1 [97]. This illustrated the activation of the oxidative/reductive stress signalling pathway (via Nrf2) in muscles [97].

### 3.3. Danio rerio (Zebrafish) Models

#### Zebrafish Models Mirrored Patient and Mice Model Cardiac and Skeletal Muscle Phenotypes, and Presented a Feasible Avenue to Facilitate High Throughput Drug and Therapeutic Target Screening

*Danio rerio* (*D. rerio*), popularly known as the zebrafish, belongs to the osteichthyan branch, which also includes mice and humans. The zebrafish *lmna* is an orthologue of human *LMNA*. The zebrafish is an established model of cardiac diseases owing to its cardiac system’s relative comparability to its mammalian counterpart, its transparency during early development, which allows for organ observation, its high fecundity and its ability to tolerate severe cardiac dysfunction that would be lethal at the foetal level in mammals [99,100]. A study by Koshimizu et al. showed that morpholino (MO) knockdown of zebrafish *lmna* resulted in slow skeletal muscle fibres damage at 24 h post-fertilization (hpf) [101]. Moreover, about half of the morphants also presented with craniofacial deformity and an increase in apoptosis in the brain and trunk [101]. Such an increase in apoptosis is further verified by impaired cell cycle progression [101]. Decreased lipid levels associated with decreased levels of zebrafish PPAR-gamma, suggestive of defects in adipocyte differentiation, were also observed [101]. Another study with MO-mediated *lmna* knockdown showed that the zebrafish lamin A/C morphants presented with blood congestion at 24 hpf, cardiac oedema, decreased heart rate, and decreased cardiac function during the period of observation (48–96 hpf) [100]. Such phenotypes are indicative of progression towards heart failure. Furthermore, some of the *lmna* morphants presented with some form of cardiac arrhythmia including atrial fibrillation, thus closely phenocopying *LMNA* mutation-caused DCM disease presentation in humans [100]. A disrupted nuclear membrane was also observed [100].

Aside from morphants, zebrafish mutant lamin A/C transgenic lines have also been generated to study striated muscle laminopathies. Two transgenic lines with cardiac-specific expression of GFP-tagged zebrafish *lmna* EDMD variants were created by Verma and Parnaik [102]. Homozygous mutant lamin A/C transgenic zebrafish have comparable fertility and longevity as WT lamin A/C transgenics up to one year old [102]. However, mutant zebrafish cardiomyocytes presented with abnormal nuclear shape, lamin aggregates (particularly the p.Q291P mutants) and an increase in heart rate at 3.5 and 5.5 d post-fertilization compared to WT [102]. Moreover, an upregulation of the expression of genes involved in cardiac regeneration was observed in whole embryo extracts from homozygous mutant lamin A/C transgenic embryos compared to WT lamin A/C transgenics [102]. In addition, PCNA, a marker for proliferation, was increased in the adult hearts of mutant transgenic lines compared to WT transgenic [102].

### 3.4. Mus musculus (Mice) Models

Out of the animal models discussed in this review, *Mus musculus* (*M. musculus*) is the species most closely related to us. The mice prelamin A shares 96.4% amino acid sequence identity with human prelamin A (Table S1). Such conservation between humans and mice lamin A/C also extends to numerous aspects of anatomy and physiology, including both species having a four-chambered heart that functions comparably. Thus, mice models provide a means to reproduce the human disease phenotype in a more closely phylogenetically related species. Various genetic modifications—knock-out, transgenesis and knock-in—have been used and yielded high fidelity in vivo striated muscle laminopathy models. 

#### 3.4.1. *Lmna* Null Mice Models Proved the Causal Link between Lamin and Human Laminopathy Phenotypes, Resulting in Lethality in the Young—But Heterozygous Mice Failed to Show the Laminopathy Phenotype

To further elucidate the importance and function of A-type lamins, *Lmna* knock-out mice were created. The lamin A/C null mice were generated using the gene trap technology [103]. No lamin A/C transcript or protein was detectable in the *Lmna*^GT−/−^ mice and both emerin and NPCs were mislocalized [103]. The *Lmna*^GT−/−^ mice presented with severe growth delay starting at seven days after birth and died at 2‒3 weeks after birth [103]. No notable phenotype was observed in the heterozygous mice [103]. Transcriptome analyses prior to onset and after the onset of phenotype in the lamin A/C null mice showed deregulation of genes involved in metabolism, adipogenesis, muscle contraction and cardiac differentiation compared to WT. Unlike in some patient samples and other striated laminopathy mouse models, there were no observed disorganized or dystrophic muscles and no fibrosis in cardiac tissues of the *Lmna*^GT−/−^ mice [38,103]. Although cell proliferation levels were comparable to WT, notable cardiac hypotrophy, which worsened with age, was observed in homozygous mice [103]. However, no functional or anatomical abnormalities (i.e., enlarged cardiac chambers/thinning of cardiac walls) were observed in homozygous hearts, except for a moderate decrease in heart rate when compared to WT [103]. Skeletal muscle was also evaluated in the homozygous mice as these animals exhibited a hunched posture and abnormal gait by 13 days of age [103]. Similar to cardiomyocytes, skeletal myocytes were hypotrophic but were neither disorganized nor dystrophic [103]. Although gonadal fat stores and brown fat were present and normal in the *Lmna*^GT−/−^ mice, subcutaneous fat was lacking [103]. Thus, adipogenic differentiation was induced in MEFs of the lamin A/C null mice and it was discovered that there was an impairment in the ability of the cells to differentiate into adipocytes when compared to induced cells from WT [103]. Metabolic parameters were also assessed and mice without lamin A/*C* become hypoglycemic and increasingly catabolic compared to WT [103]. All of these thereby illustrated the significance of lamin A/C in the context of striated muscle and fat cell differentiation, development and growth during the early stages after birth. 

Another *Lmna* null (*Lmna*^Δ/Δ^) mice was created using a different technique, Cre-mediated recombination [104]. The *Lmna*^Δ/Δ^ mice were indistinguishable from their littermates at birth [104]. However, after two weeks, *Lmna*^Δ/Δ^ mice weighed about half as much as their siblings and died by 16‒18 days of age [104]. Haematoxylin and eosin staining of skeletal muscle of 15-day-old *Lmna*^Δ/Δ^ mice showed significantly smaller muscle fibres compared to WT mice [104]. These characteristics were comparable to those observed in the *Lmna*^GT−/−^ mice [103,104]. In order to characterize and highlight the importance of each A-type lamin and its proper processing, another group of mice models was examined.

#### 3.4.2. Homozygous C-Terminal Truncated Lamin A Mice Model Recapitulated the *Lmna* Null Mice Phenotype and the Heterozygous Mice Displayed a Late-Onset Cardiac Phenotype

A very well characterized model is the *Lmna*^Δ8–11^ mice expressing a truncated lamin A that lacks its C-terminus [61,105]. It was the first (unsuccessful) attempt to create a Lamin A/C null mouse and was made by deleting exon 8 to mid-exon 11 of the *Lmna* gene [61]. The *Lmna*^Δ8–11^ mice were comparable at birth to their heterozygous and WT siblings [61]. However, significant growth retardation was noted starting at 2–3 weeks after birth and by week 3‒4, major gait defects were observed and all homozygous mice were dead by eight weeks [61]. In addition, the homozygous mice weighed considerably less than their siblings (about half their weight) [61,62]. Moreover, there was a significant increase in the organ to body weight ratio in most tissues of the *Lmna*^Δ8–11^ mice compared to WT [62]. This could be a result of increased organ function (e.g., kidneys) or a maladaptive organ enlargement (e.g., heart) [62]. Histological analyses of the cardiac and skeletal muscle tissues of *Lmna*^Δ8–11^ mice showed dystrophy, although no significant increase in creatine kinase was observed [61]. Lamin A/C transcript levels between affected (e.g., skeletal muscles) and unaffected (e.g., adipose tissues) tissues also did not seem to differ [106]. This suggested that lamin A/C levels do not contribute to the tissue-specific expression of disease phenotype in this model [106]. Furthermore, although the homozygous mice exhibited a deficit in fat tissue, analyses of metabolic parameters of these mice did not show strong lipodystrophic traits reminiscent of FPLD [61,106]. However, results from a more recent biochemical serum test hinted at potential liver dysfunction due to a significant increase in alanine aminotransferase and aspartate transaminase, which are markers for liver disease, when compared to levels found in their heterozygous and WT siblings [62]. Immunostaining of MEFs from *Lmna*^Δ8–11^ mice for lamina-associated polypeptide 2 (LAP2), Nup153 (component of NPCs) and lamin B showed misshapen nuclei and mislocalization of proteins such as lamin B and emerin, mimicking what was observed in the lamin A/C null cells of the child homozygous for the p.Y259X lamin A/C variant [46,61]. 

On the other hand, heterozygous mice (*Lmna*^Δ8–11/+^) displayed late-onset cardiomyopathy (with or without CD) with no skeletal muscle defect [107]. At 10 weeks, 62% (*n* = 18) of *Lmna*^Δ8–11/+^ mice exhibited rhythm or conduction abnormalities compared to none in WT mice (*n* = 17) [107]. Histochemical analyses of cardiac tissue also showed increased atrioventricular node fibrosis and apoptosis, and elongated nuclei in conductive cells compared to WT [107]. Moreover, aged (>50 weeks) heterozygous mice presented with significantly enlarged cardiac chambers and decreased fractional shortening indicative of both morphological and functional abnormalities [107]. However, levels of MAPK members were normal, unlike in the p.H222P EDMD mice model (to be discussed later in the review) [21,107].

#### 3.4.3. Mice Expressing Non-Farnesylated Prelamin A Only, Mature Lamin A Only or Lamin C Only Showed the Importance of Lamin A Processing and the Respective Roles of Lamins A and C in the Pathogenesis of Laminopathies

As opposed to the *Lmna*^Δ8–11^ and *Lmna*^Δ8–11/+^ mice models that express a truncated lamin A, this next mice model expresses non-farnesylated prelamin A only (*Lmna*^nPLAO/nPLAO^) [61,107,108]. These *Lmna*^nPLAO/nPLAO^ mice presented with and died because of dilated cardiomyopathy [108]. Fibroblasts from *Lmna*^nPLAO/nPLAO^ mice had increased nucleoplasmic prelamin A localization and nuclear blebs, but there was no significant change in the nuclear stiffness [108]. However, Coffinier et al. demonstrated a notable decrease in nuclear stiffness in the *Lmna*^nPLAO/nPLAO^ MEFs compared to WT [109]. Overall, *Lmna*^nPLAO/nPLAO^ mice died prematurely (all males died by 44 weeks and females by 80 weeks) [108]. Cardiac assessment and histology showed a dilated left ventricle, decreased left ventricular ejection fraction, and mild to moderate fibrosis [108]. Moreover, as in cardiomyopathy, qRT-PCR revealed decreased myosin heavy-chain alpha (*Myh6*) expression and increased myosin heavy-chain beta (*Myh7*) expression [108]. It was suggested that non-farnesylated prelamin A acted as a poison or that non-farnesylated prelamin A is less effective than mature lamin A, leading to haploinsufficiency [108]. Both hypotheses have been suggested for the development of dilated cardiomyopathy in humans [64,110,111]. Although inhibition of farnesyltransferase has been shown to improve the progeroid phenotypes, the accumulation of non-farnesylated prelamin A may result in the development of cardiomyopathy [108,112].

In contrast to the *Lmna*^nPLAO/nPLAO^ mice, mature lamin-A-only-expressing mice (*Lmna*^LAO^) were normal, healthy, did not present any discernible pathological phenotype and had comparable survival (>24 months) to WT mice [109]. Lamin A was predominantly located in the nuclear rim, similar to WT [109]. However, a significant increase in the frequency of misshapen nuclei and nuclear blebs was observed in fibroblasts from the *Lmna*^LAO^ mice compared to WT [109]. Closer inspection of the blebs showed a honeycomb staining pattern for both lamin A and LAP2β, but no difference was observed between the nuclear stiffness of WT and *Lmna*^LAO^ mice [109]. This suggested that more localized lamina structural defects such as blebbing do not necessarily result in significant global nuclear mechanical defects and subsequent clinical onset of laminopathy [109]. 

A mouse model that lacks lamin A but expresses lamin C only (*Lmna*^LCO^) was comparable to WT with no overt physiological and cellular defects (in either cardiac or skeletal muscles) over two years of observation [56]. Analyses of primary fibroblasts from *Lmna*^LCO^ embryos also showed that lamin C, emerin and LAP2 localization was normal and that there was no significant alteration in nuclear lamina structure [56]. However, a notable increase in nuclear strain in response to mechanical stress was observed in *Lmna*^LCO^ cells when compared to WT cells [56]. This showed that lamin A is essential to the nuclear lamina’s ability to respond to mechanical stress. 

Although the mice models discussed so far provide valuable information to determine and understand the respective roles of lamins A and C and their importance as a whole, such models do not recapitulate the genetic context in most laminopathy patients. The knock-in and transgenic mice allow for better mimicking of the diseased state found in patients.

#### 3.4.4. *Lmna* Knock-In and Transgenic Mice Further Established the Genotype and Phenotype Correlation and Revealed Several Signalling Pathways Involved in the Development of Myopathies Caused by *LMNA* Mutations

The p.N195K lamin A/C variant is associated with DCM-CD in humans [52]. The homozygous p.N195K lamin A/C mice (*Lmna*^N195K/N195K^) had a mild delay in weight gain beginning at four weeks but appeared to be otherwise healthy until an acute period of deterioration followed by death at 12–14 weeks after birth [86]. The homozygous males died before the homozygous females [86]. The heterozygous p.N195K mice had a comparable life span to the WT [86]. No hallmark signs of muscular dystrophy were observed in *Lmna*^N195K/N195K^ mice but mild muscular degeneration and cardiac chamber dilatation coupled with thinning of the chamber walls were noted in cardiac tissues [86]. In line with the restructuring occurring in cardiac tissues in DCM, there was an increase in interstitial fibrosis and upregulation of foetal genes such as the atrial natriuretic peptide (ANP) and brain natriuretic peptide (BNP) [86]. Furthermore, *Lmna*^N195K/N195K^ mice presented with a significant decrease in fractional shortening compared to their siblings at eight weeks, and the condition worsened with age [86]. Close monitoring and examination of the heart further revealed that the homozygous p.N195K mice presented with episodes of severe bradycardia and conduction defects, which caused death in these animals [86]. Characterization of the ventricular myocyte action potential showed longer duration associated with an increase in late sodium (Na^+^) current in the homozygous mice compared to WT [113]. Primary mouse embryonic fibroblasts from homozygous mice also showed abnormal nuclear shape, mislocalization of emerin and Nup154 (a component of NPCs), disruption of the cytoskeleton and seemingly enlarged endoplasmic reticulum and mitochondria [86]. The muscle-specific intermediate filament desmin, which connects the contractile apparatus to the sarcolemmal cytoskeleton, the organelles and the nucleus, was disorganized and less expressed in cardiac tissues from the *Lmna*^N195K/N195K^ mice [86]. In addition, connexin (CX) 40 and 43, proteins involved in gap junctions responsible for pulse relay, were mislocalized, with slightly lower amounts of CX40 in mutant atria compared to WT [86]. These therefore show that *LMNA* mutations impact the physiology of the entire cell, including the plasma membrane organization, through their effect on an intermediate filament protein like desmin. Mis-expression of the transcription factor Hf1b/Sp4, which is important in cardiac cell differentiation, particularly for the development of the cardiac conduction system, was also noted [86,114]. This model therefore mimicked the presentation of the disease in humans and demonstrated the involvement of gap junction proteins and Hf1b/Sp4 in the aberrant cardiac electrical relay that is seen in *LMNA*-related DCM-CD.

Another model for DCM-CD is transgenic mice with cardiac-specific expression of the p.E82K lamin A/C variant (*Lmna*^E82K^) [115]. This variant results from an exon 1 *LMNA* mutation identified in a Chinese family with DCM-CD (atrioventricular block) [116,117]. Transgenics were comparable to their non-transgenic siblings at birth and at a young age [115]. A portion (11–16%) of transgenic mice died starting at three months old [115]. Gross morphological changes include increased heart to body weight ratio and dilation of both cardiac chambers [115]. Cardiac functional assessment also showed a decrease in heart rate and cardiac function [115]. Moreover, like in the p.H222P mice model, interstitial fibrosis and disorganized cardiac muscle cells were also observed [115,118]. The nuclear membrane was compromised, and enlarged mitochondria and sarcoplasmic reticulum were noted [115]. In addition, elevated levels of markers for hypertrophy such as BNP, actin alpha 1 and collagen III alpha 1 were observed [115]. Thus, the mutation affecting a region in the lamin A/C protein involved in forming homodimers negatively affected actin alpha 1 and collagen III alpha 1 structural proteins that are both involved in contractility, thereby potentially causing cardiac dysfunction in the p.E82K transgenic mice. Furthermore, the transgenic mice heart displayed an increase in apoptosis, which may be linked to the decrease in left ventricular function and eventual heart failure [115].

To model another type of striated muscle laminopathy, EDMD, which also oftentimes co-present with a cardiac phenotype, a transgenic mouse with cardiac-specific expression of the p.M371K lamin A variant causing EDMD in humans was created [41,119]. This variant resulted in high prenatal deaths in the transgenics [119]. Furthermore, transgenic mice live for 2‒7 weeks only [119]. Histology of cardiac tissues from transgenic mice showed cardiac lesions, pulmonary and cardiac oedema; however, unlike other models, they did not present fibrosis [119]. Immunostaining of cardiac sections showed deformed nuclei and lamin A aggregates that were colocalized with lamin B1 [119]. Similar to some patient tissues and other mouse models, electron microscopy of p.M371K mice cardiac samples also showed chromatin clumps and lipid pseudo-inclusions in the deformed nuclei [119]. However, the fibres containing these nuclei were generally neither apoptotic nor necrotic [119]. These results illustrated that, when overexpressed in the heart, an EDMD mutant acted in a dominant negative manner, mimicking human DCM presentation.

To model another EDMD laminopathy associated with arrhythmia, the *LMNA* mutation substituting A to C at nucleotide position 665, resulting in the lamin A/C protein p.H222P variant, was expressed in mice [41]. The homozygous p.H222P knock-in mice model (*Lmna*^H222P/H222P^) presented with skeletal and cardiac phenotypes mimicking the human disease presentation. All homozygous mice died at 13 months [118]. Analyses of tissue samples showed dilatation of the ventricles, accompanied by thinning of the ventricular wall, cardiomyocyte degeneration and fibrosis [118]. Locomotor behaviour was also impaired and further examination of muscle tissues showed a wide range of abnormalities [118]. A significant increase in nuclear localization of phosphorylated-Smad2/3 was observed in *Lmna*^H222P/H222P^ mice compared to their heterozygous and WT siblings [118]. These proteins are part of the TGF-β signalling pathway, which is involved in the fibrotic process [118]. Moreover, as in the human disease presentation, there was a difference between the severity and temporal onset of the phenotype between genders in the H222P mice [118,120,121]. Investigating this difference, Arimura et al. showed an increase in the nuclear accumulation of androgen receptors and their co-factors (serum response factor [SRF] and four-and-a half LIM protein-2 (FHL2)) in male cardiac tissues from patients with DCM associated with *LMNA* mutations [121]. Furthermore, castration or flutamide (an antagonist of androgen receptors) treatment improved the symptoms of the diseased p.H222P male mice, while testosterone administration to female p.H222P mice exacerbated their phenotype [121]. Therefore, phenotype severity of *LMNA*-related DCM may depend on sex hormone levels and inhibition of the androgen receptor‒SRF‒FHL2 complex may provide a potential therapeutic benefit [121]. The MAPK kinase pathway (ERK 1/2, p38α, JNK branches) and its downstream targets were also involved in striated muscle laminopathies in both patients and *Lmna*^H222P/H222P^ mice cardiac and/or skeletal muscle samples [21,67,122]. Inhibition of the ERK 1/2, JNK, or p38α signalling pathways rescued the cardiac and/or skeletal muscle phenotypes of the *Lmna*^H222P/H222P^ mice [67,122,123,124,125,126]. Similarly, a combination of angiotensin II converting enzyme (ACE) and MEK 1/2 inhibitors improved the cardiac phenotype in the p.H222P mice [127]. Further analyses of the *Lmna*^H222P/H222P^ mice implicated the AKT/mTor and WNT/β-catenin pathways [67,128,129]. Indeed, inhibition of mTor and activation of WNT/β-catenin signalling improved the cardiac function in the homozygous p.H222P mice [67,128,129]. Impaired autophagy has also been observed in the cardiac tissues of both the human patients and the *Lmna*^H222P/H222P^ mice and the inhibition of mTor improved autophagy in the p.H222P mice hearts [128]. Gap junction architecture was disturbed in the *Lmna*^H222P/H222P^ mice as seen in the *Lmna*^N195K/N195K^ mice [86,129]. However, the localization of emerin was normal in striated muscles [118]. Therefore, this EDMD mouse model revealed new pathways that underlie striated muscle laminopathies, and that can potentially be targeted for therapeutic intervention.

A third EDMD mice model expressing another variant, the truncated p.K32del lamin A/C variant (*Lmna*^ΔK32/ΔK32^), which results in severe EDMD or L-CMD in patients, was generated [88,89]. The *Lmna*^ΔK32/ΔK32^ mice presented with major growth retardation and died three weeks after birth due to severe metabolic deficiencies associated with the repression of SREBF-1 [130]. Moreover, similar to what was observed in *C. elegans*, this lamin A/C protein variant is present in low amounts, suggesting its instability compared to the WT lamins [87,130]. The p.K32del variant also remained nucleoplasmic compared to the WT lamin A/C, which was found in both the nucleoplasm and nuclear rim [130]. On the other hand, the heterozygous p.K32del lamin A/C mice (*Lmna*^ΔK32/+^) progressively developed DCM and died at between 35 and 70 weeks of age [65]. No phenotype difference was observed between males and females [65]. Upregulation of mRNA levels for cardiac remodelling markers (*Nppb*, *Myh7*) was noted at 10 weeks (a time point that precedes notable phenotype) [65]. Immunostaining and electron microscopy revealed overt nuclear aberrations in the cardiomyocytes of heterozygous mice, particularly towards the end stage of the disease [65]. In the heterozygous mice heart tissue, total lamin A/C protein (WT and variant) levels were deemed to be 50% lower than the amounts in age-matched (10 weeks, 30 weeks) WT mice, but this discrepancy was abrogated at 57 weeks when an increase in total lamin A/C was observed [65]. The restoration of total cardiac lamin A/C in older *Lmna*^ΔK32/+^ mice was caused by the impairment of the ubiquitin-proteasome system [65]. This system was determined to be involved in the degradation of lamin A/C variant proteins in the hearts of younger *Lmna*^ΔK32/+^ mice [65]. Thus, the results suggested that the p.K32del lamin A/C variant acted as a toxic molecule and its increasing amount with age was detrimental to the *Lmna*^ΔK32/+^ mice, which eventually developed cardiomyopathy. 

However, not all mice models faithfully mimic the human phenotype. The p.L530P lamin A/C variant was discovered in a patient with autosomal dominant EDMD [44]. Heterozygous p.L530P knock-in mice were comparable to their WT siblings up to six months old [131]. However, the homozygous p.L530P mice (*Lmna*^L530P/L530P^) started presenting phenotype reminiscent of HGPS at 4–6 days old and died within 4–5 weeks [131]. Mild to intermediate degeneration, hypoplasia and atrophy of the cardiac and skeletal muscles were observed in *Lmna*^L530P/L530P^ mice [131]. Furthermore, there were fewer myocytes but an increase in fibrocytes in mutant tissues was observed [12]. At the cellular level, nuclear disruption was also observed in *Lmna*^L530P/L530P^ MEFs as well as a decrease in nuclear lamin A, and a mislocalization of lamin C to the cytoplasm instead of in the nucleus [131]. Nevertheless, no hallmarks of muscular dystrophy (e.g., centrally located nuclei and muscle fibres of various diameters) were noted and the mouse phenotype was consistent with progeria even though this mutation at the heterozygous state was associated with skeletal muscle laminopathy in humans [44,131].

## 4. Discussion and Conclusions

The evidence thus far demonstrates the wide array and importance of the presence and function of A-type lamins in maintaining cellular processes, as indicated by the diversity of diseases caused by *LMNA* mutations. Although *LMNA* mutations are known to cause distinct laminopathy disorders in humans, there is a large spectrum of sometimes contradictory findings obtained in cellular and animal models. One should not underestimate the fact that most mechanistic outcomes stem from models with inherent pitfalls. Differences in results between cell lines can be attributed to various reasons including the type of cell line used, the plasmid used for transfection (e.g., a difference in expression levels of transgene based on the promoter used), the presence or absence of a C or N termini reporter tag of various sizes, the duration of transient transfection, and which lamin (A or C) is being transfected [49,50,53]. Selecting a cell line based on the tissue-specific presentation of the disease (e.g., skeletal muscle cell lines for EDMD) is more appropriate because the cellular context in which the mutant transgene will be expressed corresponds to the cell type affected in the patients. The importance of the cell line selection can be seen in the transfection of the DCM mutant lamin A producing the p.E203G variant. In p.E203G lamin A-expressing HeLa cells, lamin aggregates were found but no such aggregates were observed upon expression in C2C12 cells [15,53]. Transfection of both lamins A and C maintains the endogenous stoichiometry between the two isoforms, therefore mimicking the naturally occurring state in cells. It also better recapitulates the autosomal dominant form of laminopathies in humans because double-transfected cells express both the WT lamin A/C (endogenous) and the mutated lamin A/C (transfected; exogenous). However, only lamin A has been expressed in most cellular models. Derived cells from iPSCs of patients, especially when cultured in 3D, present a promising means to model laminopathies as these cells demonstrate high fidelity in mimicking the patient cellular phenotypes [81]. Moreover, iPSCs not only allow for the derivation of various cell types, which is very useful in laminopathies affecting multiple tissue types, but can also circumvent the limitations associated with senescence in primary cells after prolonged culturing [51]. In addition, since these cells are from the patients themselves, they may help reconcile diversity in phenotypes observed in cellular and animal models. The cells can also be used for personalized therapeutic tests. However, as we progress in the use of iPSCs to model diseases and conduct clinical tests, we must consider the efficiency (currently typically low (<1%)) and methods used (via viruses or synthesized RNAs or proteins or small molecules) to generate iPSCs [132]. Moreover, iPSCs have been shown to differ from the original cells in terms of somatic mutations, copy number variations and DNA methylation [132,133]. Further investigation and understanding of such genomic changes in iPSCs will ensure not only the fidelity of the derived cells in reproducing the disease but, more importantly, their safety when administered to humans as treatment. Nevertheless, iPSCs and 3D culturing possess immense potential to model laminopathies and advance the field. 

Although cell line models are more manageable to handle and cheaper than in vivo models, both cellular and animal models are complementary when modelling a complex human disorder. The animal models enable not only the identification of the molecular mechanisms affected by the variant protein, but also frame the results in the context of a living organism as a whole with the opportunity to further probe the phenotype and molecular implications in specific and relevant organs/tissues in real time and/or post-mortem, as well as to eventually test for treatments. Differences or inconsistencies between the phenotypes of animal models of the same species can be a consequence of the respective techniques used. Such is the case in *Lmna*^Δ8–11^ mice, which were initially deemed to be null but later proven to express a C-terminal truncated lamin A, and the real *Lmna* null—the *Lmna* KO mice (*Lmna*^GT−/−^) created using gene trap technology, in which no lamin A/C transcript or protein was detectable [61,103,105]. The genetic background of the strain used can also have an effect on the phenotype [103]. Furthermore, the expression pattern of the transgene can cause a varying phenotype. Such a situation was observed in fruit flies, where a difference in viability was noted depending on whether the transgene was expressed ubiquitously, in a tissue-specific manner, or temporally induced (e.g., under heat-shock-driven expression) [96].

On the other hand, despite the immense progress afforded by both cellular and animal models, there are still differences in phenotypes between the actual human disease presentation and the models. For instance, both knock-out/knock-in mice models and transgenic zebrafish primarily presented a phenotype at the homozygous state, while in humans the conditions are predominantly autosomal dominant [61,86,102,103,108,115]. The heterozygous animals had milder or no symptoms, a different phenotype, or the disease onset was considerably delayed compared to their homozygous siblings (e.g., *Lmna*^GT−/−^ mice; *Lmna*^Δ8–11^ and *Lmna*^Δ8–11/+^ mice; *Lmna*^ΔK32/ΔK32^ and *Lmna*^ΔK32/+^ mice) [61,65,103,107,130]. In addition, some mutations result in a different laminopathy when expressed in the model organism (e.g., the *Lmna*^L530P/L530P^ mice, see above). Furthermore, in humans, not all *LMNA* mutation carriers actually present a phenotype. Some individuals bearing the same mutation as their affected family members remain asymptomatic. This is not usually the case in the published animal models (i.e., all homozygous mice presented a phenotype). Nevertheless, the fact that animal models do not perfectly recapitulate human phenotype can be partly attributed to the genetic modification used (knockdown versus knock-out or transgenesis or knock-in), species differences and external variables. Neither a knock-down (transient) nor a knock-out (permanent) animal model that seeks to significantly lower or completely abolish gene function is a blanket representative of the human disease context because most patients possess pathogenic mutations that do not abrogate *LMNA* gene function but instead result in the production of variant lamin A/C proteins. Moreover, although transgenesis enables tissue-specific expression of mutant transgenes, the results are influenced by the fact that endogenous WT lamin A/C is still being expressed in the animals. The insertion sites, number of insertions and expression levels of transgene are less defined and are usually not consistent between lines. In addition, in some studies, the phenotype in the transgenics becomes less overt or disappears after a few generations [134]. Knock-in models, on the other hand, would be the most faithful at recapitulating the genetic context of the mutation, but differences between species might influence and/or cause discrepancies between the human phenotype and animal models. Alignment (Figure S1) and percentage sequence identity (Table S1) between human prelamin A and its orthologues/paralogs in the different animal models cited show a range of sequence identity from 30% in *C. elegans* lamin-1 to 96.4% in *M. musculus*. Another example of species difference is with zebrafish, which underwent genome duplication, resulting in functional duplicates of orthologues for some human genes. Though the *LMNA* gene has one identified orthologue in zebrafish, *SYNE1*, which codes for the lamin A/C interacting partner Syne1, has two orthologues in zebrafish, *syne1a* and *syne1b*. In terms of anatomy, fruit flies, for instance, have a tubal heart and zebrafish only have two cardiac chambers. Thus, these species differences could contribute to the differences in phenotype between animal models and the actual human disease presentation. Other variables that can influence the phenotype also include the gene pool, environmental conditions, co-morbidities and technology. Humans have vast genetic diversity compared to that of laboratory animals. Co-morbidities include hypertension, myocarditis, autosomal disorders, diabetes, obesity, high cholesterol, inactivity, as well as aging and treatments such as chemotherapy. The effects of the environment/lifestyle on the organism are also important, particularly since for each human being the experience is unique, while lab animals live in unnatural and controlled standard lab settings. Lastly, the lag in our capacity to identify and/or characterize phenotypes in human and animal models limits our understanding of the scale and intricacies of the disease. 

Altogether, the insights learned from both cellular and animal models so far suggest that there is no one hypothesis that can completely explain the mechanisms underlying striated muscle laminopathies. However, the gene and/or mechanical hypotheses seem to provide an explanation for the cardiac and/or skeletal muscles phenotypes in most cases. The overlap in phenotypes between humans and models, particularly the phenotypic features common to more than one model, highlights the important and conserved pathways involved in the pathogenesis of striated muscle laminopathies. Table 1 and Table 2 highlight these affected proteins and signalling pathways. An example of such commonality due to the presence of pathogenic muscular *LMNA* mutations is the nuclear membrane disturbance(s) that compromises the structure, integrity, shape and/or size of the nucleus [15,38,46,50,51,54,73,87,90,91,96]. These changes can impair the ability of A-type lamins to interact with each other or with other proteins and/or chromatin. In turn, such disturbances affect the function and regulation of lamin A/C binding partners as well as the accessibility of certain chromatin regions, thereby affecting gene expression and overall cell function, including mechano-transduction signalling. Tissue-specific effects that present as the primary overt phenotype of an affected individual might be partly attributed to the resulting physical and chemical properties changes in the lamin A/C protein variants that negatively impact tissue-specific interactions and functions. Such vulnerabilities can result in mishandled cellular stimuli that feed back into the already compromised network, further magnifying the malignant cell response, with the most affected system(s) presenting as the dominant clinical symptom. In cases where the same mutation presents a different phenotype (e.g., symptomatic versus asymptomatic; difference in affected tissue) between individuals, such a contrast in disease presentation might be attributed to several factors including the state of zygosity, genetic background, epigenetic background and lifestyle. Some *LMNA* mutations exert their effects on a recessive mode. An example is the *LMNA* mutation resulting in the p.H222Y variant that caused severe EDMD for the homozygous patient, while both his heterozygous parents were asymptomatic [135]. Another lamin A/C variant, p.R298C, was identified in multiple families from Algeria and Morocco, where individuals carrying the mutation at the heterozygous state were unaffected or presented with EDMD or isolated cardiac disease and, at the homozygous state, predominantly presented with the neuropathic disease CMT2B1 [136,137,138,139,140]. The prevalence and presence (or absence) of a particular tissue-specific primary phenotype plus the varying onset and severity of clinical presentation in the families are probably influenced by differences in the genetic, epigenetic and lifestyle contexts.

With the advent of the CRISPR/Cas9 technology, genetic manipulation has become more accessible and more precise; thus, the modelling of human diseases can be even further improved. Creating knock-in *LMNA* mutations in more relevant cellular models would usher an improved model that can better mimic what is found in patient cells. The use of iPSCs from patients also allows for a superior simulation of the cell type and tissue-specific conditions. Moreover, since iPSCs are patient-specific, derived cells can be used to conduct personalized drug screens, therefore facilitating a personalized medicine approach to treating the disease. In terms of vertebrate in vivo models, the zebrafish, with its quick, transparent development and high fecundity, allows for mass drug screening. In addition, the determination of specific molecular signatures prior to disease onset is now more feasible, thus eventually enabling clinicians to improve predictive diagnostic capabilities that may allow for prophylactic treatments to either prevent or delay disease onset and attenuate the severity of the disease. Lastly, as evidenced by the progress in the field arising from results obtained from animal models (specifically the H222P EDMD mice), a clinical trial involving a p38 inhibitor (ARRY-371797) to treat patients with DCM caused by *LMNA* mutations is in progress. Phase 2 results showed improved functional capacity (assessed by a 6-min walking test) and cardiac function with mild to moderate adverse effects (Clinical Trials ID: NCT03439514), thus raising our hopes that it can be used to treat laminopathies in the future [141]. 

## Figures and Tables

**Table 1:** Summary of prominent and overlapping protein mislocalization in cellular and animal laminopathy models.

Mislocalized Proteins	Role	Mislocalization	Model(s)/Patient Sample(s)
1. Lap2 (α, β)	regulation of nuclear architecture	absence in nuclear poles and/or lobules, honeycomb pattern in nuclear blebs	fibroblasts from homozygous p.Y259X patient [46], *Lmna* ^LAO^ mice [109], *Lmna*^Δ8–11^ mice MEFs [61]
2. Emerin	anchorage to the cytoskeleton	cytoplasmic, concentration in one pole, sequestration within nuclear lamin foci, honeycomb pattern	fibroblasts from homozygous p.Y259X patient [46], RNAi *LMNA* knockdown in HeLa cells [48], various lamin A/C variants expressed in different cell lines [49,50], *C. elegans* [90], fibroblasts from patients with various lamin A/C mutations [51], myogenic cells derived from myopathies patients’ iPSCs [81], *Lmna*^Δ8–11^ mice [50,61], *Lmna*^N195K/N195K^ mice [86], p.K46del lamin-1 variant *C. elegans* [87]
3. Syne1	anchorage to the cytoskeleton	cytoplasmic	fibroblasts from homozygous p.Y259X patient [46]
4. B-type lamins	involved in a variety of functions including regulation of expression, mitosis, cellular senescence	absence in one pole (i.e., concentration in one pole only) and/or absence in nuclear lobules, honeycomb pattern	fibroblasts from homozygous p.Y259X patient [46], fibroblasts from patients with various lamin A/C mutations [51], myogenic cells derived from myopathies patients’ iPSCs [81], *LamC* null and Lamin-C N-terminal deleted *D. melanogaster* mutants [93], *Lmna*^Δ8–11^ mice MEFs [61], cardiac expressing p.M371K lamin A/C mice [119]
5. Nup153, Nup154	component of the nuclear pore complex	absence in nuclear poles and/or lobules, clustering	fibroblasts from homozygous p.Y259X patient [46], fibroblasts from p. R225X patient [79], RNAi *lmn-1* knockdown in *C. elegans* [83], *Lmna*^Δ8–11^ mice [61], *Lmna*^N195K/N195K^ mice [86], *LamC* null and various lamin A/C mutations expressed in *D. melanogaster* [96], *Lmna*^GT−/−^ mice [103], *Lmna*^Δ8–11^ mice MEFs [61]
6. SUMO1	post-translational modifications	sequestration within nuclear lamin foci	various lamin A/C variants expressed in Cos7 cells [38], C2C12 cells [78], *Lmna*^H222P/H222P^ mice primary myoblasts [78], *Lmna*^H222P/H222P^ mice skeletal muscle tissue [78]
7. Actin	cytoskeletal component	filament disorganization, increased nuclear localization, and decreased expression	neonatal rat ventricular myocytes expressing various mutant lamin A/C [34], *LamC* null *D. melanogaster* [96], Lamin-C N-terminal deleted and various lamin A/C mutations expressed in *D. melanogaster* [97], patient myoblasts expressing various L-CMD variants [35,36]
8. ERK ½ (phosphorylated)	involved in a variety of cellular responses	increased nuclear localization	*Lmna*^H222P/H222P^ mice, p.H222P lamin A expressing Cos7 and C2C12 cells [21]
9. Smad2/3 (phosphorylated)	TGF-β signalling pathway	increased nuclear localization	*Lmna*^H222P/H222P^ mice [118]
10. Androgen receptors, SRF -FHL2	mediating actions of androgens	nuclear accumulation	neonatal rat cardiomyocytes expressing p.H222P variant or p.R225X variant [121], *Lmna*^H222P/H222P^ mice and cardiac tissue from DCM patients [121]
11. Cx40, Cx43	gap junction proteins	Diffused pattern and decreased expression in atria	*Lmna*^N195K/N195K^ mice [86]

**Table 2:** Summary of prominent and overlapping affected proteins and signalling pathways in cellular and animal laminopathy models.

Models		Affected Proteins and Signalling Pathways	Reference(s)
**Cellular Models**	1. Fibroblasts from p.E203K DCM patient or HeLa transfected cells	↓ sumoylation	[77]
	2. Various lamin A/C variants expressed in neonatal rat ventricular myocytes	inhibition of p38 = rescue of actin and mechanical phenotype	[34]
	3. p. L530P (EDMD)	↓ binding to SREBF1	[15]
	4. Fibroblasts from p. R225X patient	inhibition of MEK/ERK 1/2 = rescue (decreased apoptosis and senescence)	[79]
	5. Myoblasts from patients expressing various L-CMD variants	deregulation of yes-associated protein and formins	[35,36]
**Animal Models**	6. Various lamin A/C variants expressed in *D. melanogaster*	↑ Nrf2, p62/SQSTM1	[97]
	7. *D. rerio lmna* morphants	↓ pparγ, ↓ PCNA	[101]
	8. *D. rerio lmna* transgenics	↑ PCNA	[102]
	9. *Lmna*^nPLAO/nPLAO^ mice	↓ Myh6, ↑ Myh7	[108]
	10. *Lmna*^N195K/N195K^ mice	reactivation of foetal genes (↑ANP, ↑ BNP), inhibition of Na^+^ channel = improve symptom, ↓ connexin 40, mis-expression of Hf1b/Sp4	[86]
	11. Mice cardiac tissue only expression of p.E82K lamin A/C variant	hypertrophy markers (↑ in BNP, actin alpha 1, collagen type III alpha 1), ↑ FAS, ↑ cytochrome C	[115]
	12. *Lmna*^H222P/H222P^ mice	TGFβ (↑ nuclear phos-Smad 2/3), ↑ MAPK members (ERK 1/2, p38, JNK),↑ AKT/mTor, ↓ WNT/β-catenin	[21,67,121,122,123,124,125,126,127,128,129]
	13. *Lmna*^ΔK32/ΔK32^ mice	repression of SREBF1	[130]
	14. Muscle tissues from patients with various myopathies	phos-lamin A/C (Ser458) by Akt	[73]
	15. *Lmna*^ΔK32/+^ mice	↑ Nppb, ↑ Myh7	[65]

## Supplementary Materials

The following are available online at https://www.mdpi.com/2073-4409/8/4/291/s1. References [142,143] are cited in the supplementary materials.

## Abbreviations

ACE	angiotensin II converting enzyme
Akt	Protein Kinase B
ANP	atrial natriuretic peptide
BNP	brain natriuretic peptide
CD	conduction defect
CMT2B1	Charcot‒Marie‒Tooth disease type 2B1
CRISPR/Cas9	Clustered regularly interspaced short palindromic repeats/CRISPR-associated protein 9
Cx 40/43	Connexin 40/43
DCM	Dilated Cardiomyopathy
DMD	Duchenne Muscular Dystrophy
EDMD	Emery‒Dreifuss Muscular Dystrophy
ERK 1/2	Extracellular signal-regulated kinases 1/2
FHL2	four-and-a half LIM protein-2
FPLD2	Familial partial lipodystrophy Dunnigan-type 2
Hf1b/Sp4	Transcription factor Sp4
HGPS	Hutchison Gilford Progeria Syndrome
hpf	hours post-fertilization
iPSCs	induced pluripotent stem cells
JNK	c-Jun N-terminal kinases
Keap1	Kelch-like ECH-associated protein 1
LAP2	Lamina-associated polypeptide 2
L-CMD	*LMNA*-related Congenital Muscular Dystrophy
LGMD1B	1B Limb-girdle muscular dystrophy
MAPK	Mitogen-activated protein kinase
MEFs	Mouse embryonic fibroblasts
MEK 1/2	Dual-specificity mitogen-activated protein kinase kinase ½
MO	Morpholino
mTor	mechanistic target of rapamycin
Myh6	myosin heavy-chain alpha
Myh7	myosin heavy-chain beta
NAT10	N-Acetyltransferase 10
NFκB	Nuclear factor kappa-light-chain-enhancer of activated B cells
NPCs	Nuclear pore complexes
Nppb	Natriuretic peptide B
Nrf2	Nuclear factor erythroid-2-related factor 2
Nup153/154	Nucleoporin 153/154
p62/SQSTM1	Sequestosome-1 (ubiquitin-binding protein p62)
PCNA	Proliferating cell nuclear antigen
PPAR-gamma	Peroxisome proliferator-activated receptors gamma
Smad 2/3	Mothers against decapentaplegic homolog 2/3
SREBF-1	Sterol regulatory element binding transcription factor 1
SRF	Serum response factor
SUMO1/2	Small ubiquitin-related modifier 1/2
syne1	Spectrin repeat-containing nuclear envelope protein 1
TF	Transcription factor
TGF-β	Transforming growth factor beta
WNT	Wingless/Integrated
