# Supplementary material for: Cellular and Animal Models of Striated Muscle Laminopathies
Source: Cells. 2019 Mar 29;8(4):291. doi: 10.3390/cells8040291 (PMC6523539; doi:10.3390/cells8040291)
Supplement: Supplementary file 1 [file cells-08-00291-s001.pdf]

```
Homo             ---------------METPSQRRATRSGAQASSTPLSPTRITRLQEKEDLQELNDRLAVY
Mus              ---------------METPSQRRATRSGAQASSTPLSPTRITRLQEKEDLQELNDRLAVY
Danio            ---------------METPGQKRSSRGGVTN---VLSPTRISRLQEKEDLSNLNDRLAVY
Drosophila       MSARRVTLNTRVSRASTSTPVGGASTSSRVGATSPTSPTRTSRQQEKEELQHLNDRLACY
Caenorhabditis   MS-SRKGTRSSRIVTLERSANSSLSNNGGDDSFGSTLLETSRLQEKDHLTSLNSRLATY


Homo             IDRVRSLETENAGLRLRITESEEVVSREVSGIKAAYEAELGDARKTLDSVAKERARLQLE
Mus              IDRVRSLETENAGLRLRITESEEVVSREVSGIKAAYEAELGDARKTLDSVAKERARLQLE
Danio            IDKVRSLEVENAGLRMRITESETEISRELSGMKAAYEAELADARKTLDSVAKERARLQLE
Drosophila       IDRMRNLENENSRLTQELNLAQDTVNRETSNLKAVYEKELAAARKLLDETAKEKAKLEID
Caenorhabditis   IDKVRQLEQENNRLQVQIRDIEVVEKKEKSNLADRFEAEKARLRRALDSAQDELAKYRIE


Homo             LSKVREEFKELKARNTKKEGDLIAAQARLKDLEALLNSKEAALSTALSEKRTLEGELHDL
Mus              LSKVREEFKELKARNTKKEGDLLAAQARLKDLEALLNSKEAALSTALSEKRTLEGELHDL
Danio            LSKVREDYKELKARNGKKEADLESALARLKDLESLLNSKDASLSTALGEKRTLEVEVRDL
Drosophila       IKRLWEENDDLKPRLDKKTKEATVAENNARLYENRYNEVNGKYNQSLADRKKFEDQAKEL
Caenorhabditis   YDAAKVEVKKLKPQVEKLERELAGAEEQALHAQSIADQSQAKQKTLQARNDKLVVENDDL


Homo             RGQVAKLEAALGEAKKQLQDEMLRRVDAENRLQTMKEELDFQKNIYSEELRETKRRHETR
Mus              RGQVAKLEAALGEAKKQLQDEMLRRVDAENRLQTLKEELDFQKNIYSEELRETKRRHETR
Danio            KAQLAKLEGSLNDAKKQLQDEMLRRVDAENRIQTLKEELEFQKNIYSEELRESKRRYESR
Drosophila       ALENERLRRQLDDLRKQLEAETLARVDLENQNQSLREELAFKDQVHTQELTETRSRRQIE
Caenorhabditis   KKQNITLRDTVEGLKKAVEDETLLRTAANNKIKALEEDLAFALQQHKGELEEVRHKRQVD


Homo             LVEIDNGKQREFESRLADALQELRAQHEDQVEQYKKELEKTYSAKLDNARQSAERNSNLV
Mus              LVEIDNGKQREFESRLADALQELRAQHEDQVEQYKKELEKTYSAKLDNARQSAERNSNLV
Danio            VVEIDSGRQQDYESKLADALTDLRNQHEEQLRIYKEEIEKTYNSKLENARSSAERNSHLV
Drosophila       ISEIDGRLSRQYEAKLQQSLQELRDQYEGQMRINREEIELLYDNEIQNLKAAANRAAQGS
Caenorhabditis   MTTYAKQINDEYQSKLQDQIEEMRAQFKNNLHQNKTAFEDAYKNKLN-------------
```

```
Homo             GAAHEELQQSRIRIDSLSAQLSQLQKQLAAKEAKLRDLEDSLARERDTSRRLLAEKEREM
Mus              GAAHEELQQSRIRIDSLSAQLSQLQKQLAAKEAKLRDLEDSLARERDTSRRLLAEKEREM
Danio            GAAHEELQQTRVRMEGVSSQLSQLQKQLAAREAKIRELEEALSRERDILRRRLEDKEKEM
Drosophila       ALATEEVRLMRTKIDGLNAKLQNLEDTNAGLNARIRELENLLDTERQRHNQYIASLEAEL
Caenorhabditis   -AARERQEEAVSEAIHLRARVRDLETSSSGNASLIERLRSELDTLKRSFQEKLDDKDARI

Homo             AEMRARMQQQLDEYQELLDIKLALDMEIHAYRKLLEGEEERLRLSPSPTSQR--------
Mus              AEMRARMQQQLDEYQELLDIKLALDMEIHAYRKLLEGEEERLRLSPSPTSQR--------
Danio            AEMRQRMQQQLDEYQELLDIKLALDMEISAYRKLLEGEEERLRLSPSPPPARGVTVTRSS
Drosophila       QRMRDEMAHQLQEYQGLMDIKVSLDLEIAAYDKLLCGEERRLNIESPGRPTTDSGISSNG
Caenorhabditis   AELNQEIERMMSEFHDLLDVKIQLDAELKTYQALLEGEEERLNLTQEAPQNTS-------

Homo             -------------SRGRASSHSSQTQGGGSVTKKRKLESTESR----SSFSQHARTSGRV
Mus              -------------SRGRASSHSSQSQGGGSVTKKRKLESSESR----SSFSQHARTSGRV
Danio            GSGSHTRVVQSSTSRTSSGSAKKRRLNDNDSDASSVVGGTVTR----TRISQQASASGRV
Drosophila       SHLTASASSRSGRVTPSGRRSATPGISGSSAVKRRRTVIDESEDRTLSEYSVNAAAKGDL
Caenorhabditis   ---------VHHVSFSSGGASAQRGVKRRRVVDVNGEDQDIDYLN--RRSKLNKETVGPV

Homo             AVEEVDEEGKFVRLRNKSNEDQSMGNWQIKRQNGDDPLLTYRFPPKFTLKAGQVVTIWAA
Mus              AVEEVDEEGKFVRLRNKSNEDQSMGNWQIRRQNGDDPLMTYRFPPKFTLKAGQVVTIWAS
Danio            TVDEVDLEGKFVRLNNKSDQDQSLGHWQVKRQIGSGTPIVYKFPPKFNLKAGQTVTIWAA
Drosophila       EIIEADVEGRFIKLHNKGTEEINLTGWQLTRIAGDE-ELAFKFSRGSKVLGGASVTIWSV
Caenorhabditis   GIDEVDEEGKWVRVANNSEEEQSIGGYKLVVKAGNK-EASFQFSSRMKLAPHASATVWSA

Homo             GAGATHSPPTDLVWKAQNTWGCGNSLRTALINSTGEEVAMRKLVRSVTVVED--DEDEDG
Mus              GAGATHSPPTDLVWKAQNTWGCGSSLRTALINSTGEEVAMRKLVRSLTMVEDNEDDDEDG
Danio            GAGGTHSPPSDLVWKTQNSWGSGDLFQTTLISSSGEEMAMRKVTRTLFQDEE--------
Drosophila       DAGTAHDPPNNLVMKKK--WPVANSMRSVLANADKEDVASYDRVR---------------
Caenorhabditis   DAGAVHHPPEVYVMKKQQ-WPIGDNPSARLEDSEGDTVSS--------------------
```

```
Homo            DDLLHHHHGSHCSSSGDPAEYNLRSRTVLCGTCGQPADKASASGSGAQVGGPIS-SGSSA
Mus             EELLHHHRGSHCSGSGDPAEYNLRSRTVLCGTCGQPADKAAG-GAGAQVGGSIS-SGSSA
Danio           ------DDEMAAHSTCGDSEYNLRSRTVLCGSCGQPSDRNSSCVSASSGVSSASRSFSSG
Drosophila      --------------------ANVSSHTSRHRSSGTP---------------STGFTLGSG
Caenorhabditis  ----------------------------------------------------ITVEFSES

Homo            SSVTVTRSYRSVGGSGGGSFGDNLVTRSYLLGNSSPRTQSPQNCSIM----
Mus             SSVTVTRSFRSVGGSGGGSFGDNLVTRSYLLGNSSPRSQSSQNCSIM----
Danio           GGGGLTEAFVSPSHFIVSNDKPRQVCTVCVEGTFVCLLNSITLWLVSLFFE
Drosophila      AGSTGVRSLFSLLF-------------------------------------
Caenorhabditis  SDPSDPADRCSIM--------------------------------------
```

Supplementary Figure 1: Amino acid sequence alignment of human (*Homo sapiens*; Uniprot ID: P02545) prelamin A and its orthologues in mouse (*Mus musculus*; Uniprot ID: P48678) and zebrafish (*Danio rerio*; Uniprot ID: A0A2R8QPG3), and its paralogues in fruit fly (*Drosophila melanogaster*; Uniprot ID: Q03427) and worm (*Caenorhabditis elegans*; Uniprot ID: Q21443) [142]. Highlighted amino acids (a.a) correspond to affected positions cited in this review. Amino acid in **red** refers to the lamin C specific variant affecting Arginine [R] (Serine [S] for lamin A) at position 571. Alignment was performed using Clustal Omega (https://www.ebi.ac.uk/Tools/msa/clustalo/) [143].

Supplementary Table 1: Percent amino acid sequence identity between the lamin A/C orthologues/paralogues relative to *H. sapiens* prelamin A. Values were obtained following amino acid sequence alignment using Clustal Omega (https://www.ebi.ac.uk/Tools/msa/clustalo/) [143].

Species	% Sequence Identity
M. musculus	96.4
D. rerio	66.83
D. melanogaster	37.65
C. elegans	30.31
